# Maternal Dietary Deficiencies in Folic Acid and Choline Change Metabolites Levels in Offspring after Ischemic Stroke

**DOI:** 10.3390/metabo14100552

**Published:** 2024-10-16

**Authors:** Faizan Anwar, Mary-Tyler Mosley, Paniz Jasbi, Jinhua Chi, Haiwei Gu, Nafisa M. Jadavji

**Affiliations:** 1 College of Osteopathic Medicine, Midwestern University, Glendale, AZ 85308, USA; faizan.anwar@midwestern.edu (F.A.); mtmosley@stanford.edu (M.-T.M.); 2 Department of Human Biology, Stanford University, Stanford, CA 94305, USA; 3 College of Health Solutions, Arizona State University, Phoenix, AZ 85004, USA; jasbi@therio.me (P.J.); jinhua.chi@asu.edu (J.C.); haiweigu@asu.edu (H.G.); 4 Systems Precision Engineering and Advanced Research (SPEAR), Theriome Inc., Phoenix, AZ 85004, USA; 5 Department of Biomedical Sciences, Southern Illinois University, Carbondale, IL 62901, USA; 6 Department of Child Health, University of Arizona, Phoenix, AZ 85004, USA; 7 Department of Neuroscience, Carleton University, Ottawa, ON K1S 5B6, Canada

**Keywords:** one-carbon metabolism, ischemic stroke, maternal nutrition, offspring, microbiome, metabolomics

## Abstract

**Background/objectives**: Ischemic stroke is a major health concern, and nutrition is a modifiable risk factor that can influence recovery outcomes. This study investigated the impact of maternal dietary deficiencies in folic acid (FADD) or choline (ChDD) on the metabolite profiles of offspring after ischemic stroke. **Methods**: A total of 32 mice (17 males and 15 females) were used to analyze sex-specific differences in response to these deficiencies. **Results**: At 1-week post-stroke, female offspring from the FADD group showed the greatest number of altered metabolites, including pathways involved in cholesterol metabolism and neuroprotection. At 4 weeks post-stroke, both FADD and ChDD groups exhibited significant disruptions in metabolites linked to inflammation, oxidative stress, and neurotransmission. **Conclusions**: These alterations were more pronounced in females compared to males, suggesting sex-dependent responses to maternal dietary deficiencies. The practical implications of these findings suggest that ensuring adequate maternal nutrition during pregnancy may be crucial for reducing stroke susceptibility and improving post-stroke recovery in offspring. Nutritional supplementation strategies targeting folic acid and choline intake could potentially mitigate the long-term adverse effects on metabolic pathways and promote better neurological outcomes. Future research should explore these dietary interventions in clinical settings to develop comprehensive guidelines for maternal nutrition and stroke prevention.

## 1. Introduction

Ischemic stroke is one of the leading causes of morbidity and mortality globally. The incidence of stroke in adults has been increasing due to factors such as high blood pressure, vascular disorders, diabetes, and obesity [1]. By 2030, the number of individuals affected by stroke is predicted to increase by 3.4 million [2]. While there has been a higher prevalence of ischemic stroke in older age individuals (>65 years) [1,3], recent studies have shown that the incidence of stroke among people under 50 years of age is alarmingly increasing [3,4,5]. More importantly, it is becoming more evident that nutrition is a well-established and modifiable risk factor for vascular dysfunction leading to ischemic stroke [6,7,8].

Although previous studies have established nutrition as a modifiable risk factor for vascular dysfunction and ischemic stroke [6,7,8], logistical challenges in conducting longitudinal studies have limited research on the impact of early-life nutrition on late-life neurological outcomes. Pregnancy and early life are critical periods for neurodevelopment and lay the foundation for future brain function and health [9,10,11]. In the US, health disparities lead many women to receive inadequate vitamins and nutrients during pregnancy. For example, nearly one in three African-American women do not get enough folic acid daily [12]. The same is true for Spanish-speaking Mexican-American women; they often do not get enough folic acid [13]. Furthermore, approximately one-fourth of women in the US do not consume enough choline from their diet [14,15], and women in low-income countries often face nutritional deficiencies during pregnancy, making this a serious global health issue [16]. Despite recent attention to the potential effects of maternal dietary deficiency on early life and childhood development, the long-term impact of maternal dietary deficiencies during pregnancy on the child’s future vascular health is not yet well understood. Evidence suggests that maternal nutrition plays an important role in disease onset and outcome later in life [17,18,19,20]. For example, maternal obesity during pregnancy is implicated in the onset of metabolic diseases, such as diabetes in offspring [18,19], but more life course studies are required [18]. Furthermore, it has been shown that the maternal environment plays an important role in cardiovascular risk in offspring [20]. Using animal models, many groups have shown that maternal diet (e.g., high-fat diet, and caloric restriction, etc.) can modulate cerebrovascular structure and function, leading to endothelial dysfunction, reduced smooth muscle contractility, and decreased myogenic tone, all of which significantly influence peripheral and cerebral vascular health in mouse and rat adult offspring [21,22,23]. Our group has demonstrated that maternal dietary deficiencies in folic acid or choline impact offspring ischemic stroke outcomes [24,25,26]. However, the mechanisms are not yet clear; our hypothesis is that maternal diet deficient in either folic acid or choline combined with ischemic stroke will result in changes to metabolites within the gut. The aim of this study was to investigate the metabolite levels in the fecal matter of female and male offspring exposed to maternal dietary deficiencies during early neurodevelopment and ischemic stroke during adulthood. Untargeted metabolite analysis has been shown to provide some insight into changes after stroke [27], predict stroke outcomes [28], as well as generate data for biomarkers [29,30].

## 2. Materials and Methods

### 2.1. Animals and Experimental Design

**Figure 1:**
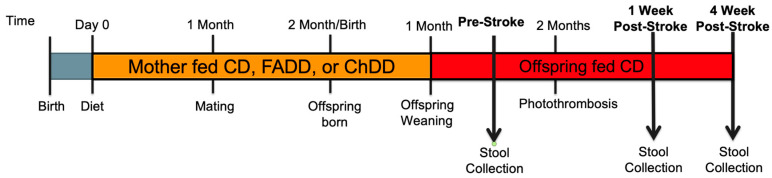
Displays the timeline of the experiment. Pregnant mothers were fed either the CD, FADD, or ChDD diet throughout the months of pregnancy and lactation until the offspring were weaned. Once the offspring were weaned, they were maintained on the CD. At 2 months of age, the offspring were subjected to ischemic stroke via the PT model. Tissue and fecal matter were collected at 1-week post-stroke and 4-week post-stroke.

All experiments were conducted in accordance with the guidelines of the Midwestern University Institutional Animal Care Users Committee (IACUC 2983). Experimental manipulations are summarized in Figure 1.

Both female and male C57/BL6J mice were obtained from Jackson Laboratories [1]. The breeding pairs produced male (*n* = 17) and female (*n* = 15) offspring. The total number of offspring used in this study was 32, consisting of 17 males and 15 females. We performed a priori sample size estimation using G*Power software (version 3.1) to determine the minimum number of animals required per group. An effect size of 0.8, a power (1-β) of 0.8, and an alpha level of 0.05 were used as parameters, resulting in a required sample size of at least 12 animals per group. The final sample size of 32 animals (17 males and 15 females) was chosen to ensure statistical power and account for possible experimental variations.

Two-month-old female mice were habituated for seven days before they were placed on either control (CD), folic acid (FADD, folic acid: 0.3 mg/kg), or choline-deficient diets (ChDD, choline bitrate: 300 mg/kg) [24,25,31] (Envigo, Indianapolis, IN, USA). The control diet contains the minimum amount of folic acid (2 mg/kg) and choline bitrate (1150 mg/kg). The dams were maintained on the diets 4 weeks prior to pregnancy, during pregnancy, and lactation. Once female and male offspring were weaned from their mothers, they were maintained on a CD. At 2 months of age, the offspring were subjected to ischemic stroke, using the photothrombosis model to the sensorimotor cortex. Fecal samples were collected from the same offspring at three time points: prior to the ischemic stroke, 1 week after, and 4 weeks after the stroke.

### 2.2. Photothrombosis Model for Ischemic Stroke

The photothrombosis model has previously been described [32,33]. Briefly, at 2 months of age, all female and male offspring mice were subjected to photothrombosis to induce a unilateral ischemic stroke in the sensorimotor cortex. Mice were anesthetized with isoflurane (1.5%) in a 70:30 nitrous oxide: oxygen mixture. Core body temperature was monitored with a rectal thermometer (Harvard Apparatus, Holliston, MA, USA) and maintained at 37 ± 0.2 °C using a heating blanket. 10 mg/kg of the photosensitive Rose Bengal (Sigma, St. Louis, MA, USA) dye was injected intraperitoneally 5 min prior to irradiation. A 532 nm green laser was placed 3 cm above the animal and directed to the sensorimotor cortex (mediolateral + 0.24 mm) for 15 min [24,25,34,35].

### 2.3. Fecal Preparation

Fecal samples were collected in a microfuge tube at three time points, prior to 1 and 4 weeks after ischemic stroke. The samples were stored at −80 °C until analysis.

Each fecal sample (~20 mg) was homogenized in 200 µL MeOH:PBS (4:1, *v*:*v*, containing 1810.5 μM ^13^C_3_-lactate and 142 μM ^13^C_5_-glutamic Acid) in an Eppendorf tube using a Bullet Blender homogenizer (Next Advance, Averill Park, NY, USA). Then 800 µL MeOH:PBS (4:1, *v*:*v*, containing 1810.5 μM ^13^C_3_-lactate and 142 μM ^13^C_5_-glutamic Acid) was added, and after vortexing for 10 s, the samples were stored at −20 °C for 30 min. The samples were then sonicated in an ice bath for 30 min. The samples were centrifuged at 14,000 RPM for 10 min (4 °C), and 800 µL supernatant was transferred to a new Eppendorf tube. The samples were then dried under vacuum using a CentriVap Concentrator (Labconco, Fort Scott, KS, USA). Prior to MS analysis, the obtained residue was reconstituted in 150 μL 40% PBS/60% ACN. A quality control (QC) sample was pooled from all the study samples.

### 2.4. Solutions for Experiments

Solutions used to complete the analysis included acetonitrile (ACN), methanol (MeOH), ammonium acetate, and acetic acid, all LC-MS grade, which were purchased from Fisher Scientific (Pittsburgh, PA, USA). Ammonium hydroxide was bought from Sigma-Aldrich (Saint Louis, MO, USA). DI water was provided in-house by a Water Purification System from EMD Millipore (Billerica, MA, USA). PBS was bought from GE Healthcare Life Sciences (Logan, UT, USA). The standard compounds corresponding to the measured metabolites were purchased from Sigma-Aldrich (Saint Louis, MO, USA), and Fisher Scientific (Pittsburgh, PA, USA).

### 2.5. Untargeted LC-MS Metabolomics

The untargeted LC-MS metabolomics method has been developed and used in a growing number of studies [36,37,38,39,40]. Briefly, all LC-MS experiments were performed using the Thermo Vanquish UPLC-Exploris 240 Orbitrap MS instrument (Waltham, MA, USA). Fecal samples were injected twice: 10 µL for analysis using negative ionization mode and 4 µL for analysis using positive ionization mode. Both chromatographic separations were performed in hydrophilic interaction chromatography (HILIC) mode on a Waters XBridge BEH Amide column (150 × 2.1 mm, 2.5 µm particle size, Waters Corporation, Milford, MA, USA). The flow rate was 0.3 mL/min, the auto-sampler temperature was kept at 4 °C, and the column compartment was set at 40 °C. The mobile phase was composed of Solvents A (10 mM ammonium acetate, 10 mM ammonium hydroxide in 95% H_2_O/5% ACN) and B (10 mM ammonium acetate, 10 mM ammonium hydroxide in 95% ACN/5% H_2_O). After the initial 1 min isocratic elution of 90% B, the percentage of Solvent B decreased to 40% at t = 11 min. The composition of Solvent B was maintained at 40% for 4 min (t = 15 min), and then the percentage of B gradually went back to 90% to prepare for the next injection. Using a mass spectrometer equipped with an electrospray ionization (ESI) source, we will collect untargeted data from 70 to 1050 *m*/*z*.

To identify peaks from the MS spectra, we made extensive use of the in-house chemical standards (~600 aqueous metabolites), and in addition, we searched the resulting MS spectra against the HMDB library, Lipidmap database, METLIN database, as well as commercial databases including mzCloud, Metabolika, and ChemSpider. The absolute intensity threshold for the MS data extraction was 1000, and the mass accuracy limit was set to 5 ppm. Identifications and annotations used available data for retention time (RT), exact mass (MS), MS/MS fragmentation pattern, and isotopic pattern. Thermo Compound Discoverer 3.3 software was used to process data on aqueous metabolomics. The untargeted data were processed by the software for peak picking, alignment, and normalization. To improve rigor, only the signals/peaks with CV <20% across quality control (QC) pools and the signals showing up in >80% of all the samples were included for further analysis.

### 2.6. Data Preprocessing

Raw data files generated from the mass spectrometer were converted to a matrix of peak intensities, with each row representing a sample and each column representing a metabolite feature. Quality control samples were also included in the matrix for inter-batch normalization. Raw data were normalized to the median of quality control samples.

### 2.7. Multivariate Analysis

Principal component analysis (PCA) and orthogonal partial least squares-discriminant analysis (OPLS-DA) were performed to explore the separation of different groups and identify important metabolites contributing to the separation.

To investigate metabolite differences between sex and maternal dietary groups, as well as their interactions, two-way ANOVA was conducted using GraphPad Prism 10. Significant main effects of two-way ANOVAs were followed by Tukey’s post-hoc test to adjust for multiple comparisons.

Statistical analysis was performed using R (version 4.1.1). For each metabolite, normality was assessed using the Shapiro-Wilk test, which is suitable for small to medium sample sizes and provides a good balance between sensitivity and specificity.

If the data for a metabolite followed a normal distribution (*p* > 0.05 in the Shapiro-Wilk test), independent t-tests were used to compare the means between groups. If the data did not follow a normal distribution (*p* ≤ 0.05 in the Shapiro-Wilk test), non-parametric Wilcoxon rank-sum tests were employed to compare the medians between groups. This test was chosen because it does not assume normality and is robust against outliers. To adjust for multiple testing and control the rate of type I errors, a false discovery rate (FDR) correction was performed using the Benjamini-Hochberg procedure.

Linear regression analysis was conducted to assess the correlation between metabolites and stroke outcomes. The regression models included sex, maternal diet, and their interaction terms to determine the influence of these factors on metabolite levels.

### 2.8. Pathway and Enrichment Analysis

Pathway analysis was performed using MetaboAnalyst (version 5.0). The pathway analysis involved the use of the hypergeometric test and pathway topological analysis. The *p*-values were not Bonferroni-corrected because the related hypotheses are not completely independent but instead share underlying biological mechanisms or functional relationships. Enrichment analysis was performed to identify the over-representation of metabolites in specific pathways using Fisher’s exact test.

### 2.9. Visualization

Heatmaps were generated to visualize the clustering of metabolites and samples. Pathway and enrichment analysis results were visualized using bubble plots and bar plots, respectively. The level of significance was set at *p* < 0.05 for all analyses.

## 3. Results

**Figure 2:**
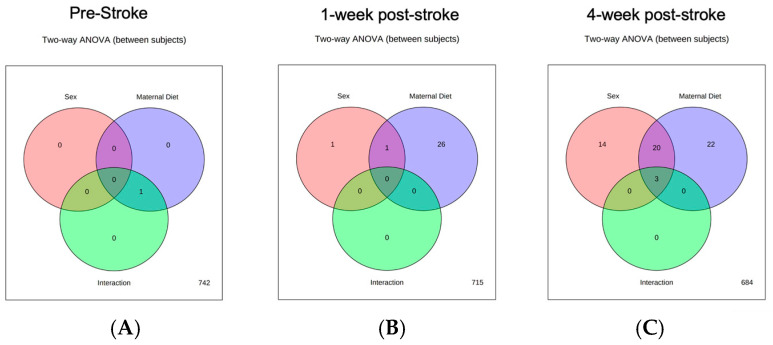
Summary of two-way ANOVA analysis demonstrating the metabolite changes based on sex, maternal diet, or interaction. (**A**) Demonstrate the difference in one metabolite pre-stroke due to a change in the maternal diet. (**B**) Demonstrate metabolite changes based on sex, maternal diet, and combined effect of both maternal diet & sex in 1-week post-stroke with no interaction. (**C**) Significant and enhanced changes in metabolites were seen at 4 weeks post-stroke based on sex, maternal diet, or combined with three metabolite changes being affected differently by sex and maternal diet.

Maternal diet deficiencies in folic acid-deficient (FADD) or choline-deficient (ChDD) diets during pregnancy and lactation significantly impacted the levels of various metabolites in offspring at both 1- and 4-week post-stroke time points. Figure 2 provides a summary of the number of metabolites altered due to maternal diet, offspring sex, or the interaction of these two variables. Notably, a higher number of significant changes were observed at the 4-week post-stroke timepoint compared to the 1-week timepoint, indicating a progressive impact of maternal diet on post-stroke recovery. Detailed information on specific metabolites with significant differences and their corresponding *p*-values are presented in Supplementary Table S1. We have listed all the metabolites that were significantly different in the supplementary data with the corresponding *p*-values for interactions between maternal diet and offspring sex, as well as the main effects of maternal diet and sex. The findings suggest that maternal dietary deficiencies may have long-term effects on the metabolic profiles of offspring following ischemic stroke, with sex-specific variations.

### 3.1. Pre-Stroke

At the pre-stroke time point, a significant difference in 4-(2-hydroxyethyl)-1-piperazineethanesulfonic acid (HEPES) level was observed between maternal diet groups (*p* = 0.00052) with female ChDD offspring having elevated levels of this metabolite (Table 1). There was also a difference between male and female offspring (*p* = 0.0087) and an interaction between maternal diet and the sex of animals (*p* < 0.0001).

### 3.2. One-Week Post-Stroke

#### 3.2.1. Interactions between Maternal Diet and Sex

There were interactions between maternal diet and offspring sex in PALDA (Table 2; *p* = 0.213), 3-Oxopalmitic Acid (*p* = 0.001), 7-Dehydrocholesterol (*p* = 0.001), Tetrahydrodeoxycorticosterone (*p* = 0.001), and Avenasterol (*p* = 0.011).

#### 3.2.2. Sex Differences

Sex differences were observed in PALDA (Table 2, *p* = 0.0307) and N6-(2-1Himidazol-4-yl) ethyl) lysine (*p* = 0.0221). Significant sex differences were followed up with pairwise comparisons. Details are listed in Table 2.

#### 3.2.3. Impact of Maternal Diet

After 1 week post-stroke we observed maternal dietary differences in fecal measurements for the following metabolites: PALDA (Table 2; *p* = 0.0006), N6-(2-1Himidazol-4-yl) ethyl)lysine (*p* = 0.001), 3-Oxopalmitic Acid (*p* = 0.017), 7-Dehydrocholesterol (*p* = 0.0001), Yakkasterone (*p* = 0.0001), Tetrahydrodeoxycorticosterone (*p* = 0.013), Cromide BEM (*p* = 0.0063), certonardosterol (*p* = 0.0001), 4betamethylzymosterol-4-carbaldehyde (*p* = 0.001), 4a,10-Dihydroxy-2,2,6a,6b,9,9,12a-heptamethyl-2,3,4,4a,6,6a,6b (*p* = 0.04), 3-dehydro-6-deoxoteasterone (*p* = 0.001), 2-Aminoethyl(2R)-3-[(1Z)-1-h(14Z)-14-Tricosen-10-one (*p* = 0.0001), 2-Aminoethyl(2R)-3-[(1Z)-1-h(14Z)-14-Tricosen-10-one (*p* = 0.0001), 1-(3,5-Dihydroxyphenyl)-2-heptadecanone (*p* = 0.0001), (R)-palmiticmonoisopropanolamide (*p* = 0.0002), (R)-palmiticmonoisopropanolamide (*p* = 0.006), and (3S,5E,7E,24R)-(6,19,19-~2~H_3_)-9,10-Secocholesta-5,7,10-trie (*p* = 0.0001). Significant maternal diet differences in metabolites were followed up with pairwise comparisons. Details are listed in Table 2.

### 3.3. Four-Week Post-Stroke

#### 3.3.1. Interactions between Maternal Diet and Sex

There was an interaction between maternal diet and offspring sex for the following metabolites, HEPES (Table 3, *p* = 0.032), 1-(4-Aminobutyl)urea (*p* = 0.0015), PRIMA-1 (*p* = 0.0005), N-lauroylglycine (*p* = 0.0036), N~6~,N~6~-Dimethyllysine (*p* = 0.0088), Misoprostol (*p* = 0.032), Gly-Leu (*p* = 0.003), Chamazulene (*p* = 0.02), Esmolol (*p* = 0.013), Dodecylethanolamide (*p* = 0.038), Creatine (*p* = 0.01), C2 Dihydroceramide (*p* = 0.003), BAR501 (*p* = 0.006), APM (*p* = 0.024), Adenine (*p* = 0.012), Acetylcadaverine (*p* = 0.0038), Acetohydroxamic acid (*p* = 0.0084), 1-PP (*p* = 0.011), (4R)-5-Hydroxy-L-leucine (*p* = 0.003), and (+)-castanospermine (*p* = 0.028).

#### 3.3.2. Sex Differences

There were sex differences in the following metabolites, Fenamole (Table 3, *p* = 0.03), n-Hexanamide (*p* = 0.0019), N-Acetyl-Lglutamic Acid (*p* = 0.051), Sphinganine (*p* = 0.02), Pyrrolidine (*p* = 0.04), Propoxur (*p* = 0.006), Phytosphingosine (*p* = 0.012), HMMNI (*p* = 0.0014), Chamazulene (*p* = 0.05), Esmolol (*p* = 0.025), and 5-Hydroxytryptophol (*p* = 0.006). There were interactions between maternal diet and sex in the following metabolites: n-Hexanamide (*p* = 0.0073), N-Acetyl-Lglutamic Acid (*p* = 0.0029), Homocitrulline (*p* = 0.0001), Sphinganine (*p* = 0.018), R-(+)-Etiracetam (*p* = 0.0005), and Pyrrolidine (*p* = 0.01). Significant sex differences between females and males were followed up with pair wise comparisons.

#### 3.3.3. Impact of Maternal Diet

At 4 weeks post-stroke there were differences in offspring metabolites, as a result of maternal diet in the following: HEPES (Table 3, *p* = 0.0072), Fenamole (*p* = 0.03), 2,3-Dihydroxypropylstearate (*p* = 0.0004), JWH 133 (*p* < 0.0001), Sphinganine (*p* = 0.053), 1-(4-Aminobutyl)urea (*p* = 0.025), Propoxur (*p* = 0.003), phytosphingosine (*p* = 0.02), N-lauroylglycine (*p* = 0.021), nicaraven (*p* = 0.003), N~6~,N~6~-Dimethyllysine (*p* = 0.049), Misoprostol (*p* = 0.036), lovastatin (*p* = 0.0081), Gly-Leu (*p* = 0.026), Chamazulene (*p* = 0.02), Esmolol (*p* = 0.0364), Dodecylethanolamide (*p* = 0.009), BAR501(*p* = 0.042), APM (*p* = 0.038), 5-Hydroxytryptophol (*p* = 0.003), and (+)-castanospermine (*p* = 0.026). Significant differences in metabolite levels as a result of maternal diet was followed up with pairwise comparisons.

## 4. Discussion

Ischemic stroke is a major health concern, with nutrition being a modifiable risk factor. We have previously demonstrated offspring from female mice deficient in either folic acid or choline have worse outcomes after stroke [25,26,41]. Metabolites are an important marker for measuring changes in the gut microbiota, which can be significant markers for different pathophysiological diseases in the body. We collected fecal samples from female and male offspring whose mothers were maintained on either FADD, ChDD, or CD. The fecal samples were collected at three time points including prior to, 1, and 4 weeks post-ischemic stroke, to measure metabolites using untargeted analyses. This untargeted metabolomics analysis revealed a wide spectrum of detrimental effects caused by stroke and maternal diet deficiencies in 3-month-old offspring. Our analysis revealed that maternal folic acid dietary deficiency has a significant impact on the microbiota of offspring after ischemic stroke. The results demonstrate that sex and maternal diet impact metabolite levels after ischemic stroke and may impact outcomes.

Prior to stroke induction, maternal diet deficiencies led to significant differences in the levels of 4-(2-hydroxyethyl)-1-piperazineethanesulfonic acid (HEPES), particularly in offspring from the ChDD group. Although the specific role of HEPES in post-stroke recovery is not fully understood, it may serve as an early indicator of altered metabolic responses due to maternal diet. As the experiment progressed to the 1- and 4-week post-stroke timepoints, these differences became more pronounced, highlighting the potential impact of maternal dietary deficiencies on metabolic adaptation to ischemic injury. The data suggest a complex interaction between maternal diet and offspring sex that may influence stroke outcomes differently at distinct recovery stages.

A total of 17 metabolite differences were detected in different diet groups at 1 week post-stroke. FADD mice had lower levels of metabolites compared to ChDD and CD, while ChDD mice had similar metabolites level to CD mice. Male mice had lower levels of metabolites at 1 week post-stroke. Most of the observed metabolites observed in lower levels are involved in various survival and developmental cellular mechanisms, including cholesterol biogenesis, cell division, macrophage polarization, and neuroprotection. Tertahydrodeoxycorticsterone, a neurosteroid, have been observed to play a role in neuroprotection and neural analgesic affects [42,43,44,45,46,47,48,49]. Another metabolite, 7-dehydrocholesterol reductase (DHCR7), is an enzyme that catalyzes 7-dehydrocholesterol (7-DHC) to cholesterol, a final step in the biogenesis of cholesterol [50,51,52]. DHCR7 mutations in humans cause a clinical autosomal recessive genetic disease, namely Smith-Lemli-Opitz syndrome (SLOS), which is characterized with multiple abnormalities including growth deficiency, intellectual disability, and frequent infections [53]. JWH 133 activates cannabinoid 2 receptors in reduced ovarian ischemia-reperfusion injury due to its antioxidant and anti-inflammatory effects [54,55,56,57]. JWH-133 showed anti-obesity effects that ameliorated pro-inflammatory M1 macrophage polarization through the Nrf2/HO-1 pathway [58]. The lower levels of the involved metabolites seem to have effects on neuroprotective mechanisms during neurodevelopment in early age as well as have effects on the immune systems and fatty acid mechanism. Dysfunction in such metabolites can also signal growth deficiency and make them more prone to infections. However, the mechanism of the effects remains unclear and requires additional investigation.

Metabolite levels were significantly lower compared to the 1-week time point but returned to normal levels by the 4-week time point. Female mice were more adversely affected, showing lower levels of all metabolites at the 4-week post-stroke time point compared to their pre-stroke levels. Mice on either ChDD or FADD were both at lower levels as compared to mice of CD. Homocitrulline levels were lower in both genders on the FADD diet, which plays a role in cell viability and mitochondrial function in menadione-treated astrocytes [59]. N-acetyl-glutamic acid, Noradrenaline, and Creatine, which play roles in liver ureagenesis, cognitive processes, and cellular metabolism essential for cognitive abilities, respectively, were found in lower levels [60,61,62,63]. Other metabolites, such as Dehydrocostus, which have antibacterial activity by inhibiting LPS-induced production of proinflammatory mediators, were found at lower levels [64]. Metabolites, which are indicators of central nervous system health and play protective roles, were detected at lower levels in females with both FADD and ChDD diets. Some of the metabolites are (+)-Castanospermine, involved in reducing inflammation in the brain, 5-Hydroxytryptophol, responsible for eliciting cerebro-arterial contractions [65,66], lower levels of acetylcadaverine is markedly found Alzheimer’s Disease [67], and BAR501 is responsible for reversing intestinal inflammation and shifts activation of intestinal macrophages reducing expression of inflammatory genes [68]. C2 Dihydroceramide associated with increased apoptosis in human leukemia HL-60 cells [69], and Gly-Leu, Glycine acts as a neurotransmitter in central nervous system; antioxidant, anti-inflammatory, cryoprotective, and immunomodulatory in peripheral and nervous tissues and leucine essential amino acid used in ATP generation, protein synthesis, tissue regeneration, and metabolism [70,71].

Overall, after 4 weeks post-stroke, it is strongly evident that female offspring that come from mothers maintained on FADD or ChDD have more changes in their metabolites compared to males. The changes are magnified at the four-week timepoint despite being on CD compared to 1-week post-stroke. The abnormal metabolite levels suggest that there is significant inflammation found both in brain and gut. It is indicated that the body’s anti-inflammatory and defense system is also highly affected. Interestingly, markers for Alzheimer’s disease, cancers, and gut and brain inflammation were found to be changed suggesting that these subjects might experience an early physical deterioration and hindered development. In a previous study, there was evidence of phenotypical behavioral issues in mice despite any changes in the ischemic size of the brain infarct. This suggests the activation of multiple pathophysiological mechanisms that have long lasting effects on a developing body. This is the first study to report changes in fecal sample metabolites after changes in maternal diet and ischemic stroke. More work is required, as the mechanism and molecular pathway are not clear. Possible next steps may include sequencing samples to determine changes in DNA. The present study provides insights that maternal dietary deficiencies in folic acid, when combined with ischemic stroke, impact offspring metabolites in a sex-dependent manner.

Although this study utilized a well-established mouse model to investigate the impact of maternal dietary deficiencies on offspring metabolite profiles, caution must be exercised when extrapolating these findings to humans. Physiological and metabolic differences between species may influence the extent to which the observed effects apply to human populations. However, the fundamental biological pathways affected by folic acid and choline deficiencies, such as one-carbon metabolism and lipid metabolism, are conserved across mammals. As a result, the insights gained from this study can serve as a foundation for future clinical research to evaluate whether similar mechanisms are present in humans. Ultimately, well-designed human studies are needed to confirm the relevance of these findings and their potential implications for maternal nutrition and offspring health.

Based on the findings of this study, specific dietary recommendations include ensuring adequate intake of folic acid and choline during pregnancy and lactation [72,73,74]. Folic acid can be supplemented through prenatal vitamins or by increasing the consumption of leafy green vegetables, legumes, and fortified cereals. Choline intake can be enhanced by incorporating foods such as eggs, lean meats, and dairy products [75,76,77]. These nutrients play a vital role in one-carbon metabolism and lipid metabolism, processes that are critical for neurodevelopment and the prevention of adverse outcomes following ischemic events. Therefore, healthcare providers should encourage pregnant and lactating women to meet the recommended dietary allowances for these nutrients to support optimal health outcomes in their offspring.

This study presents several strengths and weaknesses that merit consideration. One of the primary strengths is the use of a well-established animal model, which allows for controlled experimentation and direct observation of the effects of maternal dietary deficiencies on offspring. Additionally, the comprehensive application of untargeted metabolomics provides a broad understanding of metabolic changes and facilitates the identification of multiple metabolites involved in neurodevelopment and recovery following ischemic stroke. Furthermore, the inclusion of both male and female offspring enables the investigation of sex-based differences in response to dietary deficiencies, a factor often overlooked in similar studies.

However, there are also limitations to this research. The findings may not be fully generalizable to human populations due to physiological and metabolic differences between species. While the study had an adequate sample size for initial findings, a larger sample could enhance statistical power and allow for more definitive conclusions regarding variability in responses. Lastly, the focus on short-term outcomes, specifically at 1- and 4 weeks post-stroke, necessitates further research to assess the long-term effects of maternal dietary deficiencies on offspring health and recovery.

## 5. Conclusions

Overall, our findings show that maternal dietary deficiencies in folic acid or choline result in long-lasting alterations in the metabolite profiles of offspring post-ischemic stroke, with distinct sex-specific responses. This underscores the importance of ensuring adequate maternal nutrition during pregnancy and lactation to support optimal neurodevelopment and post-stroke recovery in offspring. Future research should explore the mechanisms underlying these changes and evaluate the efficacy of nutritional interventions as potential therapeutic strategies.

## Figures and Tables

**Table 1:** The impact of maternal dietary deficiencies in folic acid or choline on offspring fecal metabolite levels prior to ischemic stroke and summary of two-way ANOVA results.

Metabolite	Sex of Mice	CD	FADD	ChDD	*p*-Values Interaction Sex Maternal Diet
HEPES	Females	5.56 ± 0.04	5.29 ± 0.02	6.41 ± 0.22	0.01
	Males	5.68 ± 0.20	5.39 ± 0.04	5.41 ± 0.03	0.994
					0.00052

Values are expressed as the mean ± SEM of 5 to 7 mice per group. Abbreviations: CD: Control diet; ChDD: choline-deficient diet; FADD: folic acid-deficient diet.

**Table 2:** The impact of maternal dietary deficiencies in folic acid or choline on offspring fecal metabolite levels 1-week post-ischemic stroke and summary of two-way ANOVA results.

Metabolite	Sex of Mice	CD	FADD	ChDD	*p*-Values Interaction Sex Maternal Diet
PALDA	Female	5.80 ± 0.193	4.32 ± 0.07	5.41 ± 0.25	0.0213
	Male	5.04 ± 0.27	4.65 ± 0.19	4.69 ± 0.14	0.0307
					0.0006
N6-(2-1Himidazol-4-yl)ethyl)lysine	Females	5.05 ± 0.03	4.84 ± 0.03	5.04 ± 0.03	n.s.
	Males	4.97 ± 0.02	4.83 ± 0.02	4.97 ± 0.03	0.0221
					0.0001
3-Oxopalmitic Acid	Female	4.87 ± 0.01	4.79 ± 0.08	4.86 ± 0.04	0.001
	Male	5.56 ± 0.13	5.15 ± 0.11	5.01 ± 0.14	n.s.
					0.017
7-Dehydrocholesterol	Female	6.27 ± 0.04	4.464 ± 0.01	6.087 ± 0.10	0.0001
	Male	5.35 ± 0.19	5.352 ± 0.19	5.459 ± 0.13	n.n.
					0.0001
Yakkasterone	Female	6.05 ± 0.16	4.89 ± 0.26	6.097 ± 0.10	n.s.
	Male	6.21 ± 0.35	5.01 ± 0.13	6.23 ± 0.10	n.s.
					0.0001
Tetrahydrodeoxycorticosterone	Female	6.49 ± 0.38	6.58 ± 0.33	4.68 ± 0.16	0.001
	Male	6.26 ± 0.08	5.53 ± 0.08	6.45 ± 0.06	n.s.
					0.013
Avenasterol	Female	6.36 ± 0.11	4.64 ± 0.04	6.08 ± 0.10	0.011
	Male	5.86 ± 0.28	5.50 ± 0.30	6.17 ± 0.09	n.s.
					n.s.
4betamethylzymosterol-4-carbaldehyde	Female	6.52 ± 0.06	5.01 ± 0.04	6.44 ± 0.12	n.s.
	Male	6.16 ± 0.36	5.69 ± 0.26	6.59 + 0.12	n.s.
					0.001
4a,10-Dihydroxy-2,2,6a,6b,9,9,12a-heptamethyl-2,3,4,4a,6,6a,6b	Female	6.71 ± 0.09	5.12 ± 0.20	6.713 ± 0.13	n.s.
	Male	6.38 ± 0.43	5.88 ± 0.40	6.907 ± 0.07	n.s.
					0.004
3-dehydro-6-deoxoteasterone	Female	6.14 ± 0.16	4.87 ± 0.09	5.83 ± 0.15	n.s.
	Male	5.96 ± 0.21	5.48 ± 0.26	6.01 ± 0.03	n.s.
					0.0001
2-Aminoethyl(2R)-3-[(1Z)-1-h(14Z)-14-Tricosen-10-one	Female	6.18 ± 0.19	4.89 ± 0.11	5.74 ± 0.30	n.s.
	Male	5.71 ± 0.21	5.16 ± 0.13	5.96 ± 0.081	n.s.
					0.0001
1-Piperidinocyclohexanecarbonitrile	Female	6.41 ± 0.18	5.75 ± 0.12	6.69 ± 0.14	n.s.
	Male	6.28 ± 0.15	5.70 ± 0.06	6.52 ± 0.11	n.s.
					0.0001
1-(3,5-Dihydroxyphenyl)-2-heptadecanone	Female	6.13 ± 0.07	4.66 ± 0.07	6.00 ± 0.13	n.s.
	Male	6.23 ± 0.40	4.82 ± 0.07	6.30 ± 0.14	n.s.
					0.0001
(R)-palmiticmonoisopropanolamide	Female	6.46 ± 0.09	3.81 ± 0.03	6.10 ± 0.31	0.05
	Male	5.78 ± 0.48	5.15 ± 0.61	6.49 ± 0.12	n.s.
					0.0002
(22S)-22-hydroxycampest-4-en-3-one	Female	6.81 ± 0.16	4.73 ± 0.02	6.53 ± 0.18	0.0099
	Male	6.23 ± 0.37	6.00 ± 0.41	6.40 ± 0.21	n.s.
					0.006
(14Z)-14-Tricosen-10-one	Female	6.81 ± 0.16	4.84 ± 0.04	6.44 ± 0.39	0.0004
	Male	6.24 ± 0.36	6.58 ± 0.12	6.02 ± 0.21	n.s.
					0.0208
(3S,5E,7E,24R)-(6,19,19-~2~H_3_)-9,10-Secocholesta-5,7,10-trie	Female	5.92 ± 0.09	4.35 ± 0.02	5.98 ± 0.09	n.s.
	Male	5.78 ± 0.32	5.01 ± 0.26	5.93 ± 1.00	n.s.
					0.0001

Values are expressed as the mean ± SEM of 5 to 7 mice per group. Statistical significance was defined as *p* < 0.05, *p* < 0.01, and *p* < 0.001. n.s.: not significant (*p* > 0.05). Abbreviations: CD: Control diet; ChDD: choline-deficient diet; FADD: folic acid-deficient diet.

**Table 3:** The impact of maternal dietary deficiencies in folic acid or choline on offspring fecal metabolite levels 4 weeks post-ischemic stroke and summary of two-way ANOVA results.

Metabolite	Sex of Mice	CD	FADD	ChDD	*p*-Values Interaction Sex Maternal Diet
HEPES	Females	5.27 ± 0.03	5.41 ± 0.02	5.50 ± 0.06	0.032
	Males	5.39 ± 0.03	5.44 ± 0.04	5.42 ± 0.03	n.s.
					0.0072
Fenamole	Female	5.89 + 0.14	6.52 + 0.067	6.12 + 0.085	n.s.
	Male	6.46 ± 0.12	5.92 ± 0.058	6.37 ± 0.042	0.03
					0.02
2,3-Dihydroxypropylstearate	Female	6.43 ± 0.21	6.41 ± 0.12	6.32 ± 0.21	n.s.
	Male	6.24 ± 0.15	6.13 ± 0.07	6.27 ± 0.11	n.s.
					0.0004
n-Hexanamide	Female	5.89 ± 0.29	6.69 ± 0.04	6.67 ± 0.02	0.0073
	Male	7.25 ± 0.13	7.25 ± 0.35	6.57 ± 0.30	0.0019
					n.s.
N-Acetyl-Lglutamic Acid	Female	7.01 ± 0.15	7.27 ± 0.12	7.24 ± 0.10	0.0029
	Male	7.75 ± 0.11	7.18 ± 0.11	7.20 ± 0.13	0.051
					n.s.
JWH 133	Female	6.48 ± 0.29	6.68 ± 0.08	6.75 ± 0.07	n.s.
	Male	6.63 ± 0.06	6.68 ± 0.07	6.74 ± 0.05	n.s.
					<0.0001
Homocitrulline	Female	5.68 ± 0.03	6.27 ± 0.09	5.91 ± 0.07	0.0001
	Male	6.32 ± 0.08	5.72 ± 0.12	5.94 ± 0.16	n.s.
					n.s.
Sphinganine	Female	6.73 ± 0.17	7.14 ± 0.03	7.07 ± 0.03	0.018
	Male	7.16 ± 0.03	7.11 ± 0.03	7.07 ± 0.01	0.02
					0.053
R-(+)-Etiracetam	Female	5.21 ± 0.22	5.99 + 0.04	5.71 ± 0.05	0.0005
	Male	5.81 ± 0.06	5.60 ± 0.10	5.57 ± 0.09	n.s.
					n.s.
Pyrrolidine	Female	5.59 ± 0.10	6.08 ± 0.12	5.75 ± 0.12	0.01
	Male	6.14 ± 0.09	5.91 ± 0.12	5.93 ± 0.10	0.04
					n.s.
1-(4-Aminobutyl)urea	Female	4.61 ± 0.19	5.94 ± 0.08	5.44 ± 0.17	0.0015
	Male	5.40 ± 0.09	5.15 ± 0.34	5.30 ± 0.24	n.s.
					0.025
Propoxur	Female	5.31 ± 0.19	6.017 ± 0.07	5.93 ± 0.07	n.s.
	Male	5.94 ± 0.14	6.072 ± 0.09	6.18 ± 0.14	0.006
					0.003
PRIMA-1	Female	5.20 ± 0.22	6.00 ± 0.04	5.71 ± 0.05	0.0005
	Male	5.81 ± 0.06	5.60 ± 0.10	5.57 ± 0.09	n.s.
					n.s.
Phytosphingosine	Female	7.56 ± 0.21	8.14 ± 0.19	8.02 ± 0.07	0.022
	Male	8.13 ± 0.07	8.13 ± 0.018	8.15 ± 0.02	0.012
					0.02
N-lauroylglycine	Female	5.96 ± 0.13	6.46 ± 0.04	6.50 ± 0.09	0.0036
	Male	6.34 ± 0.09	6.22 ± 0.11	6.32 ± 0.04	n.s.
					0.021
Nicaraven	Female	5.24 ± 0.09	4.88 ± 0.07	4.84 ± 0.05	n.s.
	Male	5.06 ± 0.07	5.02 ± 0.10	4.94 ± 0.03	n.s.
					0.003
N~6~,N~6~-Dimethyllysine	Female	5.05 ± 0.14	5.78 ± 0.07	5.47 ± 0.13	0.0088
	Male	5.45 ± 0.12	5.31 ± 0.16	5.63 ± 0.18	n.s.
					0.049
Misoprostol	Female	6.18 ± 0.14	5.85 ± 0.08	5.69 ± 0.05	0.032
	Male	5.88 ± 0.05	5.82 ± 0.11	5.91 ± 0.10	n.s.
					0.036
Lovastatin	Female	6.86 ± 0.13	6.43 ± 0.10	6.52 ± 0.05	n.s.
	Male	6.61 ± 0.04	6.50 ± 0.05	6.53 ± 0.08	n.s.
					0.0081
HMMNI	Female	4.87 ± 0.19	5.48 ± 0.23	5.50 ± 0.05	n.s.
	Male	6.08 ± 0.19	6.03 ± 0.19	5.78 ± 0.44	0.0014
					n.s.
Gly-Leu	Female	5.38 ± 0.10	6.46 ± 0.13	5.96 ± 0.11	0.003
	Male	6.21 ± 0.15	6.07 ± 0.30	5.88 ± 0.20	n.s.
					0.026
Chamazulene	Female	5.78 ± 0.35	5.01 ± 0.01	4.929 ± 0.02	0.02
	Male	4.99 ± 0.03	4.97 ± 0.03	4.99 ± 0.02	0.05
					0.02
Esmolol	Female	6.00 ± 0.22	6.39 ± 0.07	6.43 ± 0.04	0.013
	Male	6.59 ± 0.03	6.51 ± 0.08	6.35 ± 0.06	0.025
					0.0364
Dodecylethanolamide	Female	6.38 ± 0.09	6.15 ± 0.03	6.16 ± 0.01	0.038
	Male	6.24 ± 0.02	6.23 ± 0.03	6.20 ± 0.02	n.s.
					0.009
Creatine	Female	7.21 ± 0.17	6.86 ± 0.20	7.06 ± 0.14	0.01
	Male	6.21 ± 0.22	6.98 ± 0.10	7.15 ± 0.33	n.s.
					n.s.
C2 Dihydroceramide	Female	5.55 ± 0.09	5.87 ± 0.06	5.88 ± 0.03	0.003
	Male	5.93 ± 0.04	5.73 ± 0.13	5.84 ± 0.05	n.s.
					n.s.
BAR501	Female	4.61 ± 0.06	5.15 ± 0.04	4.96 ± 0.09	0.006
	Male	5.06 ± 0.08	4.99 ± 0.11	4.97 ± 0.15	n.s.
					0.042
APM	Female	5.59 ± 0.26	6.42 ± 0.07	6.21 ± 0.10	0.024
	Male	6.16 ± 0.12	6.13 ± 0.14	6.14 ± 0.13	n.s.
					0.038
Adenine	Female	6.51 ± 0.14	7.04 ± 0.20	6.91 ± 0.21	0.012
	Male	7.39 ± 0.16	6.76 ± 0.19	7.00 ± 0.15	n.s.
					n.s.
Acetylcadaverine	Female	5.31 ± 0.01	5.80 ± 0.16	5.42 ± 0.05	0.0038
	Male	5.58 ± 0.07	5.44 ± 0.06	5.38 ± 0.03	n.s.
					0.041
Acetohydroxamic acid	Female	7.31 ± 0.08	7.75 ± 0.07	7.48 ± 0.09	0.0084
	Male	7.83 ± 0.12	7.53 ± 0.11	7.73 ± 0.17	n.s.
					n.s.
5-Hydroxytryptophol	Female	5.32 ± 0.14	6.02 ± 0.07	5.93 ± 0.07	n.s.
	Male	5.94 ± 0.19	6.07 ± 0.09	6.18 ± 0.14	0.006
					0.003
1-PP	Female	5.61 ± 0.08	6.07 ± 0.08	5.79 ± 0.10	0.011
	Male	6.14 ± 0.13	5.85 ± 0.12	6.01 ± 0.16	n.s.
					n.s.
(4R)-5-Hydroxy-L-leucine	Female	5.91 ± 0.16	6.66 ± 0.10	6.20 ± 0.13	0.003
	Male	6.64 ± 0.13	6.25 ± 0.25	6.37 ± 0.08	n.s.
					n.s.
(+)-castanospermine	Female	5.05 ± 0.29	5.94 ± 0.08	5.59 ± 0.10	0.028
	Male	5.60 ± 0.08	5.59 ± 0.10	5.69 ± 0.14	n.s.
					0.026

Values are expressed as the mean ± SEM of 5 to 7 mice per group. Statistical significance was defined as *p* < 0.05, *p* < 0.01, and *p* < 0.001. n.s.: not significant (*p* > 0.05). Abbreviations: CD: Control diet; ChDD: choline-deficient diet; FADD: folic acid-deficient diet.

## Data Availability

All relevant data can be found within the article and Supplementary Materials.

## Supplementary Materials

The following supporting information can be downloaded at: https://www.mdpi.com/article/10.3390/metabo14100552/s1, Table S1: One-week post-stroke Tukey’s pairwise comparison summary determining the impact of maternal dietary deficiencies in folic acid or choline on offspring fecal metabolite levels, Table S2: Four-week post-stroke Tukey’s pairwise comparison summary determining the impact of maternal dietary deficiencies in folic acid or choline on offspring fecal metabolite levels.
