# Supplementary material for: Maternal Dietary Deficiencies in Folic Acid and Choline Change Metabolites Levels in Offspring after Ischemic Stroke
Source: Metabolites. 2024 Oct 16;14(10):552. doi: 10.3390/metabo14100552 (PMC11509810; doi:10.3390/metabo14100552)
Supplement: Supplementary file 1 [file metabolites-14-00552-s001.zip › metabolites-3165360-supplementary.pdf]

Supplementary Data

Table S1. One-week post-stroke Tukey's pairwise comparison summary determining the impact of maternal dietary deficiencies in folic acid or choline on offspring fecal metabolite levels.

	**Female**				**Male**		
**Metabolite**	**CD vs. FADD**	**CD vs. ChDD**	**ChDD vs. FADD**		**CD vs. FADD**	**CD vs. ChDD**	**ChDD vs. FADD**
PALDA	0.002	n.s.	0.0045		n.s.	n.s.	n.s.
N6-(2-1Himidazol-4-yl)ethyl)lysine	0.0002	n.s.	0.0003		0.0044	n.s.	0.0002
3-Oxopalmitic Acid	n.s.	n.s.	n.s.		< 0.001	< 0.001	n.s.
7-Dehydrocholesterol	< 0.001	< 0.001	n.s.		n.s.	n.s.	n.s.
Yakkasterone	0.0013	n.s.	0.001		0.003	n.s.	0.0001
Tetrahydrodeoxycorticosterone	n.s.	0.0002	0.0001		n.s.	n.s.	0.0276
Avenasterol	0.001	n.s.	0.0003		n.s.	n.s.	n.s.
4betamethylzymosterol-4-carbaldehyde	0.0004	n.s.	0.0007		n.s.	n.s.	0.0004
4a,10-Dihydroxy-2,2,6a,6b,9,9,12a-heptamethyl-2,3,4,4a,6,6a,6b	0.0034	n.s.	0.003		n.s.	n.s.	0.0354
3-dehydro-6-deoxoteasterone	0.0003	n.s.	0.0043		n.s.	n.s.	n.s.
2-Aminoethyl(2R)-3-[(1Z)-1-h(14Z)-14-Tricosen-10-one	0.0008	n.s.	0.0016		n.s.	n.s.	0.0306
1-Piperidinocyclohexanecarbonitrile	0.0085	n.s.	0.0002		0.0003	n.s.	0
1-(3,5-Dihydroxyphenyl)-2-heptadecanone	0.003	n.s.	0.0001		0.0009	n.s.	n.s.
(R)-palmiticmonoisopropanolamide	0.004	n.s.	0.0004		n.s.	n.s.	0.0017
(22S)-22-hydroxycampest-4-en-3-one	0.0002	n.s.	0.0006		n.s.	n.s.	n.s.
(14Z)-14-Tricosen-10-one)	0.0002	n.s.	0.0018		n.s.	n.s.	n.s.
(3S,5E,7E,24R)-(6,19,19-~2~H_3_)-9,10-Secocholesta-5,7,10-trie	0.0255	n.s.	0.007		0.0001	n.s.	< 0.001

Values are expressed as mean ± SEM. Statistical significance was defined as *p < 0.05*, **p < 0.01**, and *$\mathbf{p < 0.001}$. *n.s.*: not significant (p > 0.05). Abbreviations: CD: Control diet; ChDD: choline-deficient diet; FADD: folic acid-deficient diet.

Table S2. Four-week post-stroke Tukey's pairwise comparison summary determining the impact of maternal dietary deficiencies in folic acid or choline on offspring fecal metabolite levels.

	Female				Male		
**Metabolite**	**CD vs. FADD**	**CD vs. ChDD**	**ChDD vs. FADD**		**CD vs. FADD**	**CD vs. ChDD**	**ChDD vs. FADD**
2,3-Dihydroxypropylstearate	n.s.	n.s.	0.0062		0.0163	n.s.	0.0115
n-Hexanamide	0.0277	0.0307	n.s.		n.s.	n.s.	n.s.
N-Acetyl-Lglutamic Acid	n.s.	n.s.	n.s.		0.0096	0.0127	n.s.
JWH 133	n.s.	n.s.	n.s.		n.s.	n.s.	n.s.
Homocitrulline	0.0004	n.s.	0.0265		0.0008	0.0349	
Sphinganine	0.0024	0.0111	n.s.		n.s.	n.s.	n.s.
R-(+)-Etiracetam	0.0001	0.0089	n.s.		n.s.	n.s.	n.s.
Pyrrolidine	0.0094	n.s.	n.s.		n.s.	n.s.	n.s.
1-(4-Aminobutyl)urea	0.0001	0.0114	n.s.		n.s.	n.s.	n.s.
Propoxur	0.0012	0.0042	n.s.		n.s.	n.s.	n.s.
PRIMA-1	0.0001	0.0088	n.s.		n.s.	n.s.	n.s.
Phytosphingosine	0.0012	0.0083	n.s.		n.s.	n.s.	n.s.
N-lauroylglycine	0.0014	0.0007	n.s.		n.s.	n.s.	n.s.
Nicaraven	0.0029	0.0011	n.s.		n.s.	n.s.	n.s.

N~6~,N~6~-Dimethyllysine	0.0014	n.s.	n.s.		n.s.	n.s.	n.s.
Misoprostol	0.0424	0.0019	n.s.		n.s.	n.s.	n.s.
Lovastatin	0.003	0.0203	n.s.		n.s.	n.s.	n.s.
HMMNI	n.s.	n.s.	n.s.		n.s.	n.s.	n.s.
Gly-Leu	0.0003	0.0448	n.s.		n.s.	n.s.	n.s.
Chamazulene	0.0037	0.0015	n.s.		n.s.	n.s.	n.s.
Esmolol	0.0355	0.0204	n.s.		n.s.	n.s.	n.s.
Dodecylethanolamide	0.0018	0.0009	n.s.		n.s.	n.s.	n.s.
Creatine	n.s.	n.s.	n.s.		0.0355	0.0091	n.s.
C2 Dihydroceramide	0.009	0.0062	n.s.		n.s.	n.s.	n.s.
BAR501	0.0005	0.0225	n.s.		n.s.	n.s.	n.s.
APM	0.0017	0.018	n.s.		n.s.	n.s.	n.s.
Adenine	n.s.	n.s.	n.s.		n.s.	n.s.	n.s.
Acetylcadaverine	0.0008	n.s.	0.007		n.s.	n.s.	n.s.
Acetohydroxamic acid	0.0176	n.s.	n.s.		n.s.	n.s.	n.s.
5-Hydroxytryptophol	0.0034	n.s.	n.s.		n.s.	n.s.	n.s.
1-PP	0.0181	n.s.	n.s.		n.s.	n.s.	n.s.
(4R)-5-Hydroxy-L-leucine	0.0034	n.s.	n.s.		n.s.	n.s.	n.s.
(+)-castanospermine	0.0011	0.0443	n.s.		n.s.	n.s.	n.s.
Fenamole	0.0003	n.s.	0.0174		0.0032	n.s.	0.0207

Values are expressed as mean ± SEM. Statistical significance was defined as *p < 0.05*, **p < 0.01**, and ***p < 0.001**. *n.s.*: not significant (p > 0.05). Abbreviations: CD: Control diet; ChDD: choline-deficient diet; FADD: folic acid-deficient diet
